# Fatigue and lack of vigor’s as a frequent work stress among financial workers in Indonesia

**DOI:** 10.3389/fpubh.2025.1563563

**Published:** 2025-09-17

**Authors:** Rofikoh Rokhim, Bagus Takwin, Ray Wagiu Basrowi, Dewi Soemaryani Soemarko, Andre Rahadian, Maria Ekowati, Kristin Samah, Nila Djuwita F. Moeloek

**Affiliations:** ^1^Faculty of Economics and Business, Universitas Indonesia, Jakarta, Indonesia; ^2^Caucus of Indonesian Mental Health Care Community (Kaukus Masyarakat Peduli Kesehatan Jiwa), Jakarta, Indonesia; ^3^Faculty of Psychology, Universitas Indonesia, Jakarta, Indonesia; ^4^Occupational Medicine Study Program, Department of Community Medicine, Faculty of Medicine, Universitas Indonesia, Jakarta, Indonesia; ^5^Health Collaborative Center (HCC), Jakarta, Indonesia; ^6^Dentons HPRP Law Firm, Jakarta, Indonesia

**Keywords:** work stress, financial sector, negative vigor, fatigue, work-life balance, employee well-being

## Abstract

This study examines work stress in the Indonesian financial sector by analyzing the prevalence of negative vigor, work fatigue, and the imbalance between work and personal life. Using a cross-sectional survey design, data were collected from employees across various financial institutions. Bivariate analysis revealed significant associations between work stress and key risk factors, with over 20% of employees reporting loss of work spirit (negative vigor) and fatigue. The results showed that younger employees (under 40 years old) are 2.5 times more likely to experience fatigue compared to older workers. Additionally, staff-level employees exhibited higher incidences of fatigue and vigor loss. The analysis also found a significant link between poor work-life balance and elevated stress levels, highlighting the lack of boundaries between personal and professional life as a key contributor to stress. These findings mirror global trends in the financial sector, where vigor loss and fatigue are significant factors in job dissatisfaction and productivity loss. The study underscores the need for targeted mental health interventions and organizational policies to address work stress and improve employee well-being.

## Introduction

Financial services is one of the most crucial sectors in the world economy, including in Indonesia, and plays a vital role in driving economic expansion, maintaining financial stability, and facilitating capital flow. However, employees in this sector are among those who face the greatest amounts of stress related to their jobs (1, 2). A 2019 survey by the American Institute of Stress found that 83% of workers in this industry said they were stressed out because of their jobs, and 60% said that their workload was a major source of stress.

Indonesia’s financial sector represents a particularly important context for studying workplace stress due to several distinctive factors. As Southeast Asia’s largest economy, Indonesia has experienced rapid financial sector growth with increasing complexity, regulatory pressures, and digital transformation demands (3). The financial sector contributes significantly to Indonesia’s GDP, employing hundreds of thousands of individuals across banking, insurance, and investment services (4). Despite its economic significance, studies examining occupational health in this sector remain limited, with most research focusing on Western contexts or manufacturing industries (5). The unique cultural dynamics of Indonesia’s work environment, characterized by hierarchical structures and collective orientation, further necessitate context-specific research as these factors may influence stress manifestations differently than in Western settings (6).

In the financial industry, stress at work can have a number of detrimental effects, such as lower productivity, higher absenteeism, and a wider economic impact (7, 8). Additionally, chronic stress is a known risk factor for the development of mental health disorders, such as anxiety and depression (9, 10), further exacerbating the challenges faced by employees in this sector. Research on high-stress professions, though in different contexts, offers valuable insights into the relationship between workplace environments and psychological wellbeing. For instance, Amorim-Gaudêncio et al. (11) found that professionals in high-stress occupations often experience compromised quality of life across multiple domains, with particular vulnerabilities in environmental satisfaction and elevated anxiety levels, patterns that may have parallels in financial sector employees exposed to prolonged work stress.

Vigor and fatigue represent opposing yet interconnected psychological states that significantly impact workplace outcomes. Vigor, characterized by high levels of energy, mental resilience, and a willingness to invest effort in work, serves as a critical component of work engagement and a predictor of productivity (12, 13). According to the Conservation of Resources theory, vigor represents a positive resource state that enables employees to effectively manage job demands and maintain performance even under pressure (14). In contrast, fatigue manifests as a state of depleted cognitive and physical resources, resulting from sustained effort expenditure without adequate recovery (15, 16).

Recent research demonstrates that these constructs operate along a continuum rather than as mere opposites, with unique antecedents and consequences (17). Vigor positively correlates with productivity metrics including task performance, creative problem-solving, and organizational citizenship behaviors, while fatigue shows robust negative associations with cognitive functioning, decision quality, and error rates (18). Importantly, the temporal relationship between these states is complex; sustained periods of high vigor without adequate recovery often lead to fatigue, creating a cyclical pattern that threatens long-term performance sustainability (19).

These dynamics are particularly pronounced in high-pressure sectors like financial services, where the combination of cognitive demands, emotional labor, and time pressure creates unique vulnerability to both vigor depletion and fatigue accumulation (20). Studies specifically examining Indonesian workforce have found that psychological factors and work environments significantly affect performance and contribute to turnover intention, highlighting the negative impacts of prolonged stress exposure (5, 21). Preventing long-term stress-related health problems, improving employee resilience, and creating a healthy work environment all depend on addressing elements that encourage vigor while reducing weariness.

The problem of workplace mental health is still not well understood in Indonesia. In the workplace, mental health is not well understood or supported, especially in the financial services industry (22). Furthermore, a lack of precise data and case studies unique to this profession frequently impedes efforts to reduce and address mental health difficulties. There is a substantial information and data gap about the Indonesian financial industry because the majority of current research concentrates on other industries or looks at these issues from an international viewpoint.

This research gap is particularly concerning given several context-specific challenges. First, Indonesia’s financial sector has undergone significant structural changes following regulatory reforms in recent years, creating novel stressors not captured in previous studies (3). Second, the sector faces acute talent retention challenges, with high turnover rates compared to the national average across industries, highlighting the need for evidence-based interventions (23). Third, cultural factors influencing help-seeking behaviors and stigma around mental health issues create barriers to implementing effective interventions without locally-relevant research (22). These contextual elements underscore the need for Indonesia-specific research rather than relying on findings from Western or even neighboring Asian countries.

In light of these difficulties, the purpose of this research is to assess how the work environment, job attributes, and mental health of workers in Indonesia’s financial services industry interact. Three main questions are addressed by this study using a cross-sectional design: (1) How common are mental health problems among workers in this industry? (2) Are work qualities and mental health outcomes significantly correlated? (3) What are some possible mitigation techniques that might be used to deal with these problems? By answering these questions, the project hopes to advance knowledge of the variables affecting mental health in the Indonesian financial industry and offer evidence-based suggestions for enhancing treatments and support for workplace mental health.

## Methods

To assess the association between the work environment, job characteristics, and mental health among employees in Indonesia’s financial services industry, this study used a cross-sectional survey design. As stated in Registration Number KEPK/UMP/01/IX/2024, the Health Research Ethics Committee of Universitas Muhammadiyah Purwokerto granted ethical approval for this study. Prior to participating, each subject gave their informed consent. Employees in the financial services industry comprising the study’s target audience were selected through purposive sampling to ensure participation from various financial institutions. Participants had to be between the ages of 20 and 55, full-time employees with at least 1 year of work experience.

The data collection utilized the Indonesian version of the New Brief Job Stress Questionnaire (SV-NBJSQ) (24), which was selected over other instruments like the Job Content Questionnaire (JCQ) due to its validation specifically for the Indonesian context. The SV-NBJSQ was developed based on the original Brief Job Stress Questionnaire (BJSQ), which has been widely used in occupational health research in Asia. According to Adi et al. (24), the Indonesian version underwent a rigorous translation and cultural adaptation process, and demonstrated acceptable reliability and validity for measuring workplace stress in Indonesian populations. The original BJSQ, on which the SV-NBJSQ is based, has shown good internal consistency with Cronbach’s alpha values ranging from 0.74 to 0.85 in previous studies (25, 26).

A mixed-mode data collection approach was implemented, with most participants completing the survey online through a secure platform, while printed questionnaires were provided for those with limited internet access or who preferred paper formats. Several strategies were employed to enhance the response rate, including institutional endorsement, strategic reminders, and clear communication about the study’s relevance to workplace well-being. Data collection occurred during September–October 2024, allowing for representation across different workload cycles in the financial sector.

Both descriptive and inferential statistics were used to examine the data. Demographic details and frequencies of various forms of work-related stress were compiled using descriptive statistics, while the associations between employment qualities, work environment, and mental health outcomes were investigated using inferential analyses including multiple regression and correlation. The SV-NBJSQ assessed nine different forms of occupational stress, including workload, conflict with coworkers, job control, and physical demands, along with 25 employment stressors such as time constraints, job uncertainty, and interpersonal connections that contribute to workplace stress.

## Results

The demographic profile of participants in this study highlights key characteristics of employees in the Indonesian financial services sector. In terms of age, the majority of respondents (47.6%) were between 30 and 39 years old, followed by 23.0% aged 40 to 49, while those aged 20 to 29 accounted for 18.7%. Younger employees under 20 years and older individuals over 59 years represented the smallest groups, with 1.0 and 0.6%, respectively. The sample also showed a gender imbalance, with 62.7% of participants being male and 37.3% female. Geographically, the largest proportion of participants were based in Central Java (24.5%), followed by East Java (20.1%) and Jakarta (17.6%), while West Java (11.1%), Banten (4.9%), and other provinces (21.8%) also contributed to the sample. Notably, the study covered all provinces except Central Papua and Mountainous Papua, ensuring a diverse regional representation (Table 1).

**Table 1:** Socio-demographic overview (*n* = 5,546).

Variable	n	%	Note
Age			
<20 y.o	56	1.0	
20–29 y.o	1,038	18.7	
30–39 y.o	2,642	47.6	
40–49 y.o	1,273	23.0	
50–59 y.o	505	9.1	
>59 y.o	32	0.6	
Sex			
Male	3,475	62.7	
Female	2071	37.3	
Location			
Central Java	1,358	24.5	There are 36 provinces, except Central Papua and Mountainous Papua.
East Java	1,115	20.1	
Jakarta	976	17.6	
West Java	618	11.1	
Banten	269	4.9	
Others	1,210	21.8	
Marriage Status			
Single	1,029	18.6	
Married	4,368	78.8	
Widowed	149	2.7	
Education			
Primary School	1	0.01	
Junior High School	3	0.1	
Senior High School	397	7.2	
Diploma	356	6.4	
Bachelor	4,182	75.4	
Master	594	10.7	
Doctoral	13	0.2	

The demographic profile of participants reveals important patterns with implications for targeted stress management in Indonesia’s financial sector. The predominance of employees aged 30–39 (47.6%) and male employees (62.7%) indicates a workforce at a career stage often balancing increasing professional responsibilities with family obligations, potentially contributing to work-life tensions. The geographical concentration in Central Java (24.5%), East Java (20.1%), and Jakarta (17.6%) reflects Indonesia’s financial industry distribution, with densely populated urban centers likely experiencing higher-pressure work environments compared to less represented regions. The high proportion of married participants (78.8%) suggests that family responsibilities may compound workplace stressors, requiring interventions that acknowledge the dual demands faced by employees. Educational data showing most employees hold Bachelor’s degrees (75.4%) indicates a highly educated workforce that may respond well to evidence-based stress management approaches, while potentially experiencing stress from educational investment and corresponding career expectations.

In terms of marital status, a significant majority of participants were married (78.8%), with 18.6% being single and 2.7% widowed, indicating a workforce largely composed of individuals managing both work and family responsibilities. Educational attainment among the participants was notably high, with 75.4% holding a Bachelor’s degree, 10.7% a Master’s degree, and 0.2% a Doctoral degree. In contrast, only a small percentage had completed education at the senior high school level (7.2%) or below, including primary school, junior high school, and diploma holders, who collectively accounted for 6.5%. This demographic overview underscores the diverse age distribution, regional representation, and high educational background of employees in the financial services sector, providing a comprehensive basis for analyzing job-related stress and mental health within this workforce (Table 2).

**Table 2:** Overview of job type (*n* = 5,546).

Variable	n	%	Mean ± SD /Median (Min–Max)
Occupational Type			
Bank Employee	3,747	67.6	
Life Insurance Employee	310	5.6	
General Insurance Employee	586	10.6	
Reinsurance Employee	19	0.3	
Multifinance Employee	103	1.9	
Securities Employee	73	1.3	
Investment Manager Employee	16	0.3	
Pension Fund Employee	8	0.1	
Appraisal Employee	0	0	
Pawn Employee	122	2.2	
Futures/Commodities Employee	8	0.1	
Financial Advisory Employee	6	0.1	
Fintech Employee	9	0.2	
Debt Collector	13	0.2	
Accountant	12	0.2	
Financial Regulator	49	0.9	
Others	465	8.4	
Occupational Status			
Permanent Employee	4,441	80.1	
Contracted Employee	1,062	19.1	
Part-Time Employee	43	0.8	
Position			
Commissioner	17	0.3	
Director	80	1.4	
Manager	1727	31.1	
Supervisor	629	11.3	
Staff	2,869	51.7	
Others	224	4.0	
Type of Institution			
Private	486	8.8	
State-Owned Enterprise	4,802	86.6	
Village-Owned Enterprise	198	3.6	
Multinational Company	7	0.1	
Combinations	53	1.0	
Work Period (Year)			11 ± 7.8 **10 (1–37)**
Work Duration/Day (Hour)			10 ± 2.3 **10 (1–24)**

Occupational characteristics reveal that bank employees (67.6%) constitute the majority of participants, indicating that interventions should prioritize banking-specific stressors such as customer-facing pressure and transaction accuracy demands. The prevalence of permanent employees (80.1%) suggests organizational interventions could have sustainable impact due to workforce stability, while the high proportion of staff-level employees (51.7%) indicates that front-line stress management deserves particular attention. The predominance of state-owned enterprises (86.6%) points to potential for standardized interventions across these institutions, leveraging their similar organizational cultures. The average 11-year work tenure alongside 10-h workdays reveals a workforce experiencing substantial cumulative stress exposure while navigating extended workdays that exceed standard hours, suggesting interventions should address both acute daily fatigue and chronic stress accumulation (Table 3).

**Table 3:** Overview of work stress according to SV-NBJSQ (*n* = 5,546).

Variable	n	%
Vigor / Vitality		
Abnormal	1,453	26.2
Normal	4,093	73.8
Anger / Irritability		
Abnormal	598	10.8
Normal	4,948	89.2
Fatigue		
Abnormal	1,082	19.5
Normal	4,464	80.5
Anxiety		
Abnormal	900	16.2
Normal	4,646	83.8
Depression		
Abnormal	321	5.8
Normal	5,225	94.2
Physical Stress Reaction		
Abnormal	692	12.5
Normal	4,854	87.5
Job Satisfaction		
Abnormal	482	8.7
Normal	5,064	91.3
Workplace Social Capital		
Abnormal	651	11.7
Normal	4,895	88.3
Work Engagement		
Abnormal	242	4.4
Normal	5,304	95.6

The prevalence of stress factors among participants provides crucial insight for intervention prioritization. With 26.2% of employees experiencing reduced vigor or vitality, 19.5% reporting fatigue, and 16.2% showing anxiety symptoms, these three conditions represent the most common manifestations of workplace stress requiring immediate attention. Physical stress reactions (12.5%) and workplace social capital challenges (11.7%) indicate additional areas for intervention. The relatively low levels of dissatisfaction (8.7%), depression (5.8%), and disengagement (4.4%) suggest that despite stress indicators, most employees maintain fundamental connection to their work. This pattern suggests early-stage intervention could prevent progression to more severe conditions, as employees appear to maintain basic job satisfaction and engagement despite experiencing energy depletion and stress symptoms. Overall, the majority of respondents exhibited normal levels of anger/irritability (89.2%) and anxiety (83.8%), indicating relatively positive mental health outcomes for a significant portion of the sample.

### Bivariate analysis for vigor

Bivariate analysis reveals that younger employees under 40 are 2.5 times more likely to experience fatigue, suggesting that organizational interventions should include specialized support for early-career professionals, such as workload management training and resilience-building programs tailored to this age group. Female employees, with 1.3 times higher fatigue risk, would benefit from gender-specific programs addressing unique stressors they face in Indonesia’s financial sector, where women often navigate both traditional gender expectations and professional demands. The regional differences indicating that employees in Java experience nearly twice the fatigue risk compared to other regions calls for regionally-tailored approaches that address the faster pace and higher demands of financial centers in densely populated areas. Married individuals showing lower fatigue risk suggests that family support may serve as a protective factor, indicating potential benefit from work-family integration programs that leverage this strength rather than treating family solely as a competing demand (Table 4).

**Table 4:** The relationship of socio-demographic factors with vigor.

Variable	Vigor				*p*	OR (95% CI)
	Abnormal		Normal			
	n	%	n	%		
Age					<0.001	2.4 (2.1–2.8)
<40 y.o	1,168	31.3	2,568	68.7		
≥40 y.o	285	15.7	1,525	84.3		
Sex					<0.001	1.7 (1.5–1.9)
Female	687	33.2	1,384	66.8		
Male	766	22.0	2,709	78.0		
Location					0.010	1.2 (1.0–1.4)
Java	1,208	26.9	3,275	73.1		
Others	245	23.0	818	77.0		
Marriage Status					<0.001	0.4 (0.4–0.5)
Married	1,007	23.1	3,361	76.9		
Not Married	446	37.9	732	62.1		
Education					0.008	1.3 (1.0–1.6)
Primary–Senior High School	128	31.9	273	68.1		
Diploma–Doctoral	1,325	25.8	3,820	74.2		

Respondents living in Java were slightly more likely to report abnormal vigor (26.9%) compared to those from other regions (23.0%), with an odds ratio of 1.2 (OR: 1.2, 95% CI: 1.0–1.4, *p* = 0.010). Marital status also influenced vigor levels, as unmarried individuals exhibited a higher prevalence of abnormal vigor (37.9%) compared to married respondents (23.1%), with an odds ratio of 0.4, indicating that married individuals were less likely to experience reduced vitality (OR: 0.4, 95% CI: 0.4–0.5, *p* < 0.001).

In terms of educational background, respondents with a primary to senior high school education had a higher prevalence of abnormal vigor (31.9%) compared to those with a diploma to doctoral degree (25.8%), with an odds ratio of 1.3 (OR: 1.3, 95% CI: 1.0–1.6, *p* = 0.008). This suggests that higher educational attainment may be associated with better vitality in the workplace (Table 5).

**Table 5:** The relationship of employment factors with vigor.

Variable	Vigor				*p*
	Abnormal		Normal		
	n	%	n	%	
Type of Occupation					0.018^cs^
Bank	988	26.4	2,759	73.6	
Insurance	242	26.4	673	73.,6	
Multifinance & Investment	55	27.5	145	72.5	
Pawn, Commodity & Advisory	23	16.9	113	83.1	
Fintech	3	33.3	6	66.7	
Debt Collector	7	53.8	6	46.2	
Regulator	23	37.7	38	62.3	
Others	112	24.1	353	75.9	
Employment Status					<0.001^cs^
Permanent Employee	1,110	25.0	3,331	75.0	
Contracted Employee	332	31.3	730	68.7	
Part-Time Employee	11	25.6	32	74.4	
Position					<0.001^cs^
Commissioner	2	11.8	15	88.2	
Director	4	5.0	76	95.0	
Manager	310	18.0	1,417	82.0	
Supervisor	154	24.5	475	75.5	
Staff	943	32.9	1926	67.1	
Others	40	17.9	184	82.1	
Type of Institution					0.366^cs^
Private	145	29.8	341	70.2	
State-Owned Enterprise	1,240	25.8	3,562	74.2	
Village-Owned Enterprise	52	26.3	146	73.7	
Multinational Company	1	14.3	6	85.7	
Combinations	15	28.3	38	71.7	
Work Period (Year)	9 (1–35)		11 (1–37)		<0.001^mw^
Work Duration/Day (Hour)	10 (2–24)		10 (1–24)		<0.001^mw^

cs: chi square; mw: mann whitney.

Staff-level employees exhibit significantly higher rates of both fatigue (24.8%) and vigor loss (32.9%) compared to management positions, likely reflecting their limited decision authority combined with high customer-facing responsibilities. For instance, tellers in regional bank branches often describe managing long queues of customers while simultaneously maintaining transaction accuracy and security protocols, with minimal ability to control their work pace. Debt collectors face particularly acute stress, with 53.8% reporting vigor loss, as they navigate challenging client interactions while meeting collection targets. As one debt collector described: “I regularly face hostile reactions while also meeting strict performance metrics, and the emotional labor is overwhelming by mid-week.” These front-line realities suggest interventions should address both the practical aspects of workload management and the psychological dimensions of emotional labor specific to each role.

Among bank and insurance employees, 26.4% reported abnormal vigor, while employees in multifinance and investment sectors showed a similar rate of 27.5%. Conversely, workers in pawn, commodity, and advisory roles had a lower prevalence of abnormal vigor at 16.9%.

Employment status also plays a critical role in vigor levels. Contracted employees experienced higher rates of abnormal vigor (31.3%) compared to permanent employees (25.0%). In terms of job positions, staff-level employees reported the highest prevalence of reduced energy at 32.9%, making them more vulnerable to stress compared to supervisors (24.5%), managers (18.0%), and directors (5.0%). Notably, 33% of staff-level employees and nearly 30% of private-sector financial workers reported reduced vigor due to workplace stress.

Institutional type showed less variation in vigor levels, with private-sector employees reporting a slightly higher prevalence of abnormal vigor (29.8%) compared to those in state-owned enterprises (25.8%) and village-owned enterprises (26.3%). Employees in multinational companies reported the lowest rates of abnormal vigor at 14.3%, suggesting a potentially more supportive work environment.

Younger employees, particularly those under 40, were 2.4 times more likely to experience reduced vigor compared to their older counterparts. Additionally, workers with shorter tenure and longer daily work hours were more prone to experiencing reduced energy levels, emphasizing the impact of work duration and experience on employee vitality. Specifically, employees with abnormal vigor had a median work period of 9 years and worked an average of 10 h per day, compared to 11 years and 10 h for those with normal vigor levels (Table 6).

**Table 6:** The relationship between stressor factors and vigor.

Variable	Vigor				*p*	OR (95%CI)
	Abnormal		Normal			
	n	%	n	%		
Quantitative Job Overload					<0.001	1.7 (1.5–1.9)
Abnormal	866	31.3	1901	68.7		
Normal	587	21.1	2,192	78.9		
Job Control					0,005	1.6 (1.2–2.3)
Abnormal	55	36.2	97	63.8		
Normal	1,398	25.9	3,996	74.1		
Interpersonal Conflict					<0.001	3.1 (2.7–3.5)
Abnormal	575	44.5	716	55.5		
Normal	878	20.6	3,377	79.4		
Suitable Job					<0.001	7.4 (6.0–9.2)
Abnormal	283	68.7	129	31.3		
Normal	1,170	22.8	3,964	77.2		
Meaningfulness at Work					<0.001	10.8 (8.6–13.4)
Abnormal	354	74.8	119	25.2		
Normal	1,099	21.7	3,974	78.3		
Support from Family & Friend					<0.001	1.9 (1.6–2.1)
Abnormal	535	35.5	972	64.5		
Normal	918	22.7	3,121	77.3		
Supervisor Support					<0.001	2.9 (2.6–3.3)
Abnormal	931	37.6	1,547	62.4		
Normal	522	17.0	2,546	83.0		
Emotional Demands					<0.001	4.4 (3.9–5.0)
Abnormal	966	43.2	1,268	56.8		
Normal	487	14.7	2,825	85.3		
Role Conflict					<0.001	2.0 (1.8–2.3)
Abnormal	930	32.6	1924	67.4		
Normal	523	19.4	2,169	80.6		
Role Clarity					<0.001	3.2 (2.1–5.0)
Abnormal	45	52.9	40	47.1		
Normal	1,408	25.8	4,053	74.2		
Career Opportunity					<0.001	4.5 (3.7–5.5)
Abnormal	264	57.8	193	42.2		
Normal	1,189	23.4	3,900	76.6		
Monetary					<0.001	2.3 (1.9–2.6)
Abnormal	356	40.8	516	59.2		
Normal	1,097	23.5	3,577	76.5		
Esteem Reward					<0.001	3.1 (2.6–3.7)
Abnormal	315	48.4	336	51.6		
Normal	1,138	23.2	3,757	76.8		
Leadership					<0.001	4.0 (3.3–4.8)
Abnormal	255	55.0	209	45.0		
Normal	1,198	23.6	3,884	76.4		
Interactional Justice					<0.001	3.8 (3.2–4.6)
Abnormal	279	53.8	240	46.2		
Normal	1,174	23.4	3,853	76.6		
Workplace Where People Compliment Each Other					<0.001	2.7 (2.3–3.2)
Abnormal	335	45.0	410	55.0		
Normal	1,118	23.3	3,683	76.7		
Workplace Where Mistakes Are Acceptable					<0.001	3.0 (2.4–3.8)
Abnormal	149	49.7	151	50.3		
Normal	1,304	24.9	3,942	75.1		
Trust with Management					<0.001	4.3 (3.4–5.4)
Abnormal	203	57.7	149	42.3		
Normal	1,250	24.1	3,944	75.9		
Preparedness for Change					<0.001	3.1 (2.7–3.6)
Abnormal	446	46.6	511	53.4		
Normal	1,007	21.9	3,582	78.1		
Respect for Individuals					<0.001	4.7 (3.9–5.5)
Abnormal	359	57.2	269	42.8		
Normal	1,094	22.2	3,824	77.8		
Fair Personnel Evaluation					<0.001	3.1 (2.7–3.7)
Abnormal	366	48.0	396	52.0		
Normal	1,087	22.7	3,697	77.3		
Diversity					<0.001	3.2 (2.7–3.8)
Abnormal	301	49.2	311	50.8		
Normal	1,152	23.3	3,782	76.7		
Career Development					<0.001	3.1 (2.6–3.7)
Abnormal	255	49.3	262	50.7		
Normal	1,198	23.8	3,831	76.2		
Work-Self Balance (Negative)					<0.001	2.9 (2.5–3.3)
Abnormal	951	36.9	1,623	63.1		
Normal	502	16.9	2,470	83.1		
Work-Self Balance (Positive)					<0.001	6.4 (5.4–7.7)
Abnormal	405	63.6	232	36.4		
Normal	1,048	21.3	3,861	78.7		

The results reveal significant associations between various workplace factors and abnormal vigor, indicating reduced energy and vitality among employees. Quantitative job overload is a notable predictor, with employees experiencing abnormal workloads being 1.7 times more likely to report reduced vigor compared to those with manageable workloads (31.3% vs. 21.1%, *p* < 0.001). Similarly, low job control is associated with a 1.6 times higher likelihood of reduced vigor (36.2% vs. 25.9%, *p* = 0.005).

Interpersonal conflict emerged as a critical factor, increasing the odds of reduced vigor by 3.1 times (44.5% vs. 20.6%, *p* < 0.001). Additionally, employees who found their jobs unsuitable were 7.4 times more likely to experience abnormal vigor (68.7% vs. 22.8%, *p* < 0.001). Lack of meaningfulness at work had the strongest association, with employees facing this issue being 10.8 times more likely to report reduced energy levels (74.8% vs. 21.7%, *p* < 0.001).

Support systems also play a significant role. Insufficient family and friend support increased the likelihood of reduced vigor by 1.9 times (35.5% vs. 22.7%, *p* < 0.001), while poor supervisor support raised the odds by 2.9 times (37.6% vs. 17.0%, *p* < 0.001). High emotional demands further increased the risk of abnormal vigor by 4.4 times (43.2% vs. 14.7%, *p* < 0.001).

Job-related stressors, such as role conflict and role clarity, were significant predictors, with abnormal clarity increasing the odds of reduced vigor by 3.2 times (52.9% vs. 25.8%, *p* < 0.001). Similarly, employees reporting limited career opportunities were 4.5 times more likely to experience reduced vigor (57.8% vs. 23.4%, *p* < 0.001).

Workplace culture also plays a critical role. Lack of leadership increased the likelihood of reduced vigor by 4.0 times (55.0% vs. 23.6%, *p* < 0.001), while low trust in management raised the odds by 4.3 times (57.7% vs. 24.1%, *p* < 0.001). A lack of respect for individuals and unfair personnel evaluations were also significant, with both factors increasing the odds of abnormal vigor by more than three times (57.2% vs. 22.2%, *p* < 0.001; 48.0% vs. 22.7%, *p* < 0.001, respectively).

Finally, negative work-self balance doubled the odds of reduced vigor (36.9% vs. 16.9%, *p* < 0.001), while positive work-self balance had a protective effect, with employees experiencing this reporting significantly better energy levels. However, a negative work-life balance increased the odds of reduced vigor by 6.4 times (63.6% vs. 21.3%, *p* < 0.001). These findings underscore the critical importance of improving workplace conditions, support systems, and job design to enhance employee well-being and vigor.

### Bivariate analysis for fatigue

The results indicate significant associations between demographic factors and the prevalence of abnormal fatigue. Individuals under the age of 40 are 2.5 times more likely to experience fatigue compared to those aged 40 and above (23.7% vs. 10.8%, *p* < 0.001). Similarly, female employees report higher fatigue levels than males, with a 1.3 times increased likelihood (22.5% vs. 17.7%, *p* < 0.001; Table 7).

**Table 7:** The relationship of socio-demographic factors with fatigue.

Variable	Fatigue				*p*	OR (95%CI)
	Abnormal		Normal			
	n	%	n	%		
Age					<0.001	2.5 (2.1–3.0)
<40 y.o	887	23.7	2,849	76.3		
≥40 y.o	195	10.8	1,615	89.2		
Sex					<0.001	1.3 (1.1–1.5)
Female	467	22.5	1,604	77.5		
Male	615	17.7	2,860	82.3		
Location					<0.001	1.9 (1.5–2.3)
Java	952	21.2	3,531	78.8		
Others	130	12.2	933	87.8		
Marriage Status					<0.001	0.5 (0.4–0.6)
Married	764	17.5	3,604	82.5		
Not Married	318	27.0	860	73.0		
Education					0.033	1.3 (1.0–1.6)
Primary–Senior High School	95	23.7	306	76.3		
Diploma–Doctoral	987	19.2	4,158	80.8		

Geographic location also plays a role, as workers in Java have a 1.9 times higher chance of experiencing fatigue compared to those in other regions (21.2% vs. 12.2%, *p* < 0.001). Marital status is inversely associated with fatigue, with married individuals showing a significantly lower likelihood of abnormal fatigue (17.5% vs. 27.0%, *p* < 0.001, OR 0.5).

Lastly, educational attainment shows a moderate association, where individuals with lower education levels (primary to senior high school) have a 1.3 times higher likelihood of fatigue compared to those with higher education (diploma to doctoral; 23.7% vs. 19.2%, *p* = 0.033). These findings underscore the influence of age, gender, location, marital status, and education on employee fatigue levels (Table 8).

**Table 8:** The relationship of employment factors with fatigue.

Variable	Fatigue				*p*
	Abnormal		Normal		
	n	%	n	%	
Type of Occupation					<0.001^cs^
Bank	865	23.1	2,882	76.9	
Insurance	101	11.0	814	89.0	
Multifinance & Investment	26	13.0	174	87.0	
Pawn, Commodity & Advisory	15	11.0	121	89.0	
Fintech	1	11.1	8	88.9	
Debt Collector	7	53.8	6	46.2	
Regulator	21	34.4	40	65.6	
Others	46	9.9	419	90.1	
Employment Status					0.854^cs^
Permanent Employee	860	19.4	3,581	80.6	
Contracted Employee	213	20.1	849	79.9	
Part-Time Employee	9	20.9	34	79.1	
Position					<0.001^cs^
Commissioner	0	0.0	17	100	
Director	2	2.5	78	97.5	
Manager	248	14.4	1,479	85.6	
Supervisor	92	14.6	537	85.4	
Staff	712	24.8	2,157	75.2	
Others	28	12.5	196	87.5	
Type of Institution					<0.001^cs^
Private	54	11.1	432	88.9	
State-Owned Enterprise	987	20.6	3,815	79.4	
Village-Owned Enterprise	29	14.6	169	85.4	
Multinational Company	1	14.3	6	85.7	
Combinations	11	20.8	42	79.2	
Work Period (Year)	9 (1–34)		10 (1–37)		<0.001^mw^
Work Duration/Day (Hour)	12 (3–24)		9 (1–24)		<0.001^mw^

cs: chi square; mw: mann whitney.

The analysis highlights several critical factors influencing fatigue among financial sector employees. Younger workers under 40 years old are 2.5 times more likely to experience work-related fatigue, primarily driven by stress. Additionally, employees based in Java face nearly double the risk of fatigue compared to those in other regions, reflecting regional stress disparities.

In terms of job roles, debt collectors report the highest fatigue prevalence, with 53.8% experiencing stress-related exhaustion. In contrast, commissioners are the only position with no significant fatigue cases, suggesting their lower exposure to job-related stressors. Among different types of institutions, employees in state-owned enterprises (SOEs) show a higher prevalence of fatigue (20.6%), while those in private companies report the lowest (11.1%).

Employment status shows no significant differences, with fatigue levels being similar across permanent (19.4%), contracted (20.1%), and part-time employees (20.9%). However, fatigue risk increases with longer working hours, as fatigued employees report a median work duration of 12 h per day, compared to 9 h among those without fatigue. Similarly, fatigued employees have a shorter average work period (9 years) compared to their non-fatigued counterparts (10 years), indicating that prolonged exposure to stress may eventually lead to adaptation or exit from stressful roles.

These findings underscore the importance of managing stress and workload, particularly for younger employees, frontline roles like debt collectors, and those working extended hours in Java and SOEs (Table 9).

**Table 9:** The relationship between stressor factors and fatigue.

Variable	Fatigue				*p*	OR (95%CI)
	Abnormal		Normal			
	n	%	n	%		
Quantitative Job Overload					<0.001	2.4 (2.1–2.7)
Abnormal	727	26.3	2040	73.7		
Normal	355	12.8	2,424	87.2		
Job Control					<0.001	2.5 (1.8–3.5)
Abnormal	57	37.5	95	62.5		
Normal	1,025	19.0	4,369	81.0		
Interpersonal Conflict					<0.001	5.6 (4.8–6.4)
Abnormal	565	43.8	726	56.2		
Normal	517	12.2	3,738	87.8		
Suitable Job					<0.001	8.8 (7.1–10.9)
Abnormal	259	62.9	153	37.1		
Normal	823	16.0	4,311	84.0		
Meaningfulness at Work					<0.001	14.9 (12–18.5)
Abnormal	340	71.9	133	28.1		
Normal	742	14.6	4,331	85.4		
Support from Family & Friend					<0.001	1.6 (1.4–1.9)
Abnormal	390	25.9	1,117	74.1		
Normal	692	17.1	3,347	82.9		
Supervisor Support					<0.001	2.7 (2.3–3.1)
Abnormal	697	28.1	1781	71.9		
Normal	385	71.9	2,683	87.5		
Emotional Demands					<0.001	6.9 (5.9–8.1)
Abnormal	825	36.9	1,409	63.1		
Normal	257	7.8	3,055	92.2		
Role Conflict					<0.001	3.2 (2.7–3.7)
Abnormal	794	27.8	2060	72.2		
Normal	288	10.7	2,404	89.3		
Role Clarity					<0.001	3.2 (2.1–5.0)
Abnormal	37	43.5	48	56.5		
Normal	1,045	19.1	4,416	80.9		
Career Opportunity					<0,001	6,7 (5,5–8,1)
Abnormal	258	56.5	199	43.5		
Normal	824	16.2	4,265	83.8		
Monetary					<0.001	3.5 (3.0–4.1)
Abnormal	348	39.9	524	60.1		
Normal	734	15.7	3,940	84.3		
Esteem Reward					<0.001	4.7 (3.9–5.6)
Abnormal	307	47.2	344	52.8		
Normal	775	15.8	4,120	84.2		
Leadership					<0.001	6.7 (5.5–8.2)
Abnormal	262	56.5	202	43.5		
Normal	820	16.1	4,262	83.9		
Interactional Justice					<0.001	7.2(5.9–8.7)
Abnormal	297	57.2	222	42.8		
Normal	785	15.6	4,242	84.4		
Workplace Where People Compliment Each Other					<0.001	4.3(3.6–5.0)
Abnormal	331	44.4	414	55.6		
Normal	751	15.6	4,050	84.4		
Workplace Where Mistakes Are Acceptable					<0.001	5.1(4.0–6.5)
Abnormal	157	52.3	143	47.7		
Normal	925	17.6	4,321	82.4		
Trust with Management					<0.001	7.2(5.7–9.0)
Abnormal	209	59.4	143	40.6		
Normal	873	16.8	4,321	83.2		
Preparedness for Change					<0.001	5.3(4.5–6.2)
Abnormal	443	46.3	514	53.7		
Normal	639	13.9	3,950	86.1		
Respect for Individuals					<0.001	8(6.7–9.5)
Abnormal	363	57.8	265	42.2		
Normal	719	14.6	4,199	85.4		
Fair Personnel Evaluation					<0.001	4.1(3.5–4.8)
Abnormal	331	43.4	431	56.6		
Normal	751	15.7	4,033	84.3		
Diversity					<0.001	5.3(4.4–6.3)
Abnormal	305	49.8	307	50.2		
Normal	777	15.7	4,157	84.3		
Career Development					<0.001	3.7(3.1–4.4)
Abnormal	224	43.3	293	56.7		
Normal	858	17.1	4,171	82.9		
Work-Self Balance (Negative)					<0.001	8.1(6.8–9.6)
Abnormal	898	34.9	1,676	65.1		
Normal	184	6.2	2,788	93.8		
Work-Self Balance (Positive)					<0.001	11.1(9.2–13.3)
Abnormal	407	63.9	230	36.1		
Normal	675	13.8	4,234	86.2		

The relationship between stressors and fatigue reveals that lack of meaningfulness at work represents the highest risk factor (OR 14.9), manifesting when employees perceive their tasks as purely transactional without connection to broader purpose. As one investment advisor explained: “Processing paperwork all day with no visibility of how my work helps clients achieve their goals makes every task feel draining.” Similarly, poor work-self balance (OR 11.1) emerges when boundaries collapse, exemplified by a bank manager who reported: “I’m expected to respond to messages until midnight, and my children now associate me with always being on my phone.” The significant impact of unsuitable job placement (OR 8.8) appears when employees’ skills misalign with their roles, as when analytically-trained staff are placed in sales positions requiring different competencies. These workplace realities highlight the need for interventions addressing both structural issues like job design and cultural factors like after-hours communication expectations.

The analysis reveals significant factors associated with work-related fatigue in financial sector employees. Quantitative job overload doubles the likelihood of fatigue, with employees experiencing abnormal workload having an odds ratio (OR) of 2.4. Low job control further exacerbates fatigue risk (OR 2.5), while interpersonal conflict poses an even greater threat, increasing fatigue risk nearly sixfold (OR 5.6).

The nature of the job plays a critical role, as employees with unsuitable job roles have an OR of 8.8 for developing fatigue, whereas the lack of meaningfulness at work leads to the highest risk, with an OR of 14.9. Emotional demands (OR 6.9) and role conflict (OR 3.2) are also significant contributors to fatigue.

Support systems significantly impact fatigue levels. Employees lacking supervisor support have a 2.7 times higher risk of fatigue, and insufficient family and friend support increases the risk by 1.6 times. Similarly, perceived interactional injustice (OR 7.2) and poor trust with management (OR 7.2) are strongly associated with higher fatigue levels.

Workplace culture also plays a critical role. In environments where mistakes are not accepted (OR 5.1) or compliments are rare (OR 4.3), fatigue prevalence rises. A lack of respect for individuals (OR 8.0) and inadequate preparedness for change (OR 5.3) further compound the risk of fatigue.

Moreover, career-related factors such as limited career development (OR 3.7), poor career opportunities (OR 6.7), and unfair personnel evaluation (OR 4.1) significantly increase fatigue risk. Finally, the inability to maintain a work-self balance poses a critical risk, with negative balance showing an OR of 8.1, while a positive imbalance yields an OR of 11.1, highlighting the critical importance of balancing professional and personal life for overall well-being.

A multivariate analysis would strengthen these findings by controlling for the potential interaction between demographic factors, particularly as age, position, and work experience often correlate. Such analysis would clarify whether the higher fatigue among younger employees remains significant when controlling for their typically lower positions in organizational hierarchies. Similarly, multivariate techniques would reveal whether gender differences in stress levels persist when controlling for position level and work tenure. Future research employing such analyses would provide more nuanced guidance for intervention design, ensuring resources target the most influential factors rather than their correlates.

## Discussion

This study sheds light on a frequently disregarded area of occupational health by presenting significant data on the prevalence and risk variables linked to work stress in Indonesia’s finance sector. Even if it seems to be less common than the global average, work stress is still a serious problem that needs to be addressed (27). In particular, the study finds that job fatigue and a decline in work spirit or negative vigor are two important markers of work-related stress that more than 20% of employees suffer. These results are consistent with research conducted globally, which has identified comparable indicators as the main causes of occupational stress in the banking sector (13, 28). Another study also found that stress levels in the banking workplace have reached critical levels, with detrimental psychological and physical effects on employees, as well as negative impacts on organizations (20). These issues ranged from anxiety and depression to maladaptive behaviors and, ultimately, job burnout. The limitations of the reviewed studies were discussed, and potential directions for future research were considered.

Indonesia’s unique work culture significantly shapes how stress manifests in the financial sector. The predominant hierarchical management style, or “bapakism,” creates stress patterns distinct from Western contexts. As noted by Mukhlis et al. (29), Indonesian workplace culture often emphasizes power distance and hierarchical relationships that can affect how employees experience and report stress. This cultural dynamic helps explain our finding that staff-level employees experience significantly higher fatigue rates (24.8%) compared to management positions. The concept of “rubber time” (jam karet) in Indonesian business culture, while offering flexibility, often translates into unpredictable working hours for financial sector employees, contributing to the poor work-self balance that our study identified as a major stressor (OR 11.1). Additionally, Indonesia’s strong collectivist orientation creates implicit expectations to participate in work-related social activities outside office hours, further blurring work-life boundaries in ways not typically seen in more individualistic Western work cultures (29).

Compared to global data, the stated prevalence of stress in Indonesia’s banking sector is noticeably lower. For example, a 2020 survey by the Financial Services Authority (OJK) revealed a 70% prevalence rate in Indonesia, whereas a Deloitte (30) global report found that 77% of financial sector professionals experienced stress. This study, on the other hand, emphasizes a lower overall prevalence while highlighting the importance of fatigue and negative vigor as important risk variables. This finding can be attributed to the fact that this study divides work stress into 9 types, and each of these types has a prevalence around 5 to 25%. These results highlight the necessity of focused interventions that deal with these problems in order to reduce stress and enhance workers’ general well-being.

Systematically compared to global studies, these findings reflect a nuanced picture. For example, Giorgi et al. (31) conducted a multicountry survey and reported that banking employees frequently experience emotional exhaustion, depersonalization, and reduced personal accomplishment, with prevalence rates exceeding 75% in some regions, particularly during the COVID-19 pandemic. Similarly, Hassard et al. (32) found through a cross-national study in the United Kingdom, Germany, and Japan that job demands, lack of control, and work–family conflict were key predictors of stress. Interestingly, the Indonesian context in this study reveals lower aggregate stress, possibly due to cultural coping mechanisms, organizational structures, or underreporting related to stigma, as noted by Chen et al. (33) in their study on Asian workplace mental health.

In Indonesia, two to three out of 10 employees in the banking sector suffer from low work morale, often known as negative vigor. This result is marginally less than global data from the Journal of Occupational Health Psychology (2020), which found that 40% of workers in the banking sector globally experience negative vigor. Even though it is less common, negative vigor is still a serious problem because it is linked to lower levels of job satisfaction and productivity. However, according to research from around the world, 20% of Indonesian financial workers suffer from fatigue. Fatigue is a crucial component of stress management techniques since it is the primary indication of work stress among employees in the financial sector worldwide, according to Deloitte (34). Applying theoretical models such as the Job Demand–Control (JDC) model (35) and the Job Demands–Resources (JD-R) model (36), the findings gain further explanatory depth. The JDC model posits that high job demands coupled with low job control predict high strain and poor health outcomes. The identification of fatigue and negative vigor aligns with the strain predicted under this framework. The JD-R model, which emphasizes the balance between job demands and available resources, also helps explain these results even if overall stress prevalence is lower, specific demands such as cognitive fatigue or emotional exhaustion become critical when resources like supervisor support or recovery opportunities are insufficient.

A study reported that poor employee morale and negative attitudes toward work might be caused by low pay, long hours, lack of motivation, understaffed and unskilled labor, high physical workload, and inadequate supervision (37). This fact is happening in the workplace of financial sector. Therefore, feedback from managers revealed that employees had limited understanding of the company’s mission and vision, and there was also a noticeable disconnect between workers and managers. Potential interventions, such as increasing employee-supervisor interactions, promoting positive behavior, offering non-monetary benefits, providing training, ensuring wage rate and employee selection consistency, and redesigning jobs, were recommended to management to improve the existing conditions.

The study also shows that young workers under 40 are especially likely to experience negative vigor and exhaustion. Compared to their elder colleagues, these workers are 2.5 times more likely to feel fatigued. This conclusion is important since the financial sector’s long-term productivity and sustainability are directly impacted by the well-being of its younger workers, who frequently makes up the majority of the workforce. Negative vigor, exhaustion, and boredom can be caused by changes in work functions or environment (38). Additionally, up to 30% of staff-level employees report experiencing weariness and vigor, suggesting that lower-level jobs may be more susceptible to stress-related issues. Specifically, in terms of vigor, this study agrees with the fact that public firms have lower level of employee stress compared to private firms (39), but that is not the case in terms of fatigue. Meta-analyses by Koutsimani et al. (40); Salvagioni et al. (41) consistently demonstrated that burnout components particularly emotional exhaustion are universal across sectors, but their expression and intensity vary by cultural, organizational, and individual factors. The relatively lower stress rates in Indonesia compared to global data may thus reflect cultural resilience or collectivistic workplace norms, which prioritize harmony and social support, acting as buffering resources, as outlined in the COR theory (14). This perspective suggests that interventions should not only target individual stressors but also enhance systemic resources that mitigate stress reactions.

Based on our findings, we propose several concrete interventions tailored to Indonesia’s financial sector context. First, financial institutions should implement mandatory “electronic sunsets” that disable work-related communications between 8 PM and 7 AM, directly addressing the work-self balance issues identified as major stressors. Second, organizations should develop age-stratified mental health programs targeting younger employees through digital platforms, acknowledging both their higher risk status (2.5 times more likely to experience fatigue) and their technological preferences. Third, financial institutions should establish formalized “pembinaan programs” that adapt traditional Indonesian mentorship approaches to specifically address stress management, leveraging cultural familiarity while targeting modern workplace challenges. Fourth, given the significantly higher stress levels among staff-level employees (32.9% experiencing vigor loss), organizations should implement rotation policies limiting customer-facing work to 4-h blocks, allowing recovery periods between high-intensity interactions. Finally, regional differences in stress prevalence suggest the need for location-specific interventions, with higher-intensity support programs in Java-based operations where fatigue risk is nearly doubled compared to other regions.

The disparity between personal and professional obligations is another important element causing stress at work in the finance industry. This result is in line with international research that identifies a major occupational health concern as the blurring of the lines between personal and work obligations (42-45). According to Deloitte (34), a lack of work-life balance is a major source of stress for workers in the financial sector globally, which can have long-term health effects and impair job performance. Improving employee retention rates and creating a better work environment require addressing this imbalance.

While this study provides valuable insights into work stress in Indonesia’s financial sector, several limitations should be acknowledged. First, despite our sampling from multiple provinces, certain regions were underrepresented, particularly eastern Indonesia, including Maluku, Papua, and surrounding islands. Otherwise, this purposive sampling introduces the risk of selection bias due to regional imbalance may affect the generalizability of findings to the entire Indonesian financial sector. The concentration of respondents in Java (over 70%) potentially overrepresents urban banking experiences while underrepresenting conditions in less developed regions where different stressors may predominate. Second, our cross-sectional design, while appropriate for prevalence assessment, limits causal inference regarding the relationships between workplace factors and stress outcomes. Third, self-report nature of the data may be influenced by cultural tendencies in Indonesian workplace contexts, where employees might underreport stress due to concerns about job security or social desirability bias, particularly in hierarchical organizations where expressing difficulty might be perceived as weakness.

Given the limitations of the current study, longitudinal studies would better capture how Indonesia’s rapidly evolving financial sector and changing work practices affect employee well-being over time. Future studies should examine successful interventions that are tailored to the particular requirements of Indonesian financial sector employees, emphasizing the development of a supportive corporate culture, better work-life balance, and mental health promotion strategies that account for Indonesia’s unique cultural context and regional diversity.

In summary, the influence of negative vigor, fatigue, and work-life imbalance cannot be disregarded, even though the prevalence of job stress in Indonesia’s banking sector may be lower than global averages. These elements have a big impact on workers’ general job happiness, productivity, and well-being. This study offers practical recommendations for organizational interventions, making the results highly relevant for practitioners and policymakers seeking to improve employee well-being and productivity.

## Conclusion

The study demonstrates the complex relationship between work-related energy and exhaustion among workers in Indonesia’s financial services industry. Employee energy levels are greatly influenced by demographic variables including age, gender, and marital status as well as occupational positions, job status, and institutional type. Women, younger workers, and those in front-line positions like debt collectors are especially susceptible to fatigue and diminished vitality. Furthermore, these problems are made worse by regional differences and workplace culture, which emphasizes the necessity of targeted interventions to enhance worker well-being.

Financial institutions in Indonesia should implement comprehensive stress management strategies with specific action points: develop age-specific intervention programs for younger employees who demonstrated 2.5 times higher risk of fatigue; create women’s professional support networks to address gender disparities; establish clear policies limiting after-hours communication to address Indonesia’s blurred work-life boundaries; institute regular workload assessments during high-stress periods; design career advancement pathways that incorporate well-being metrics alongside performance indicators; and develop evidence-based training programs customized to reflect Indonesian cultural values. Additionally, industry associations such as Asosiasi Bank Indonesia and Otoritas Jasa Keuangan should establish sector-wide mental health standards and monitoring mechanisms to ensure stress management becomes an integral part of organizational governance across Indonesia’s financial sector, thereby enhancing employee well-being while contributing to long-term institutional stability and competitiveness.

## Data Availability

The original contributions presented in the study are included in the article/supplementary material, further inquiries can be directed to the corresponding author.
